# Facilitating Granule Cell Survival and Maturation in Dentate Gyrus With Baicalin for Antidepressant Therapeutics

**DOI:** 10.3389/fphar.2020.556845

**Published:** 2020-09-02

**Authors:** Fan Zhao, Weiwei Tao, Zhiyuan Shang, Weihua Zhang, Jie Ruan, Chenyiyu Zhang, Liping Zhou, Hunter Aiello, Hezheng Lai, Rong Qu

**Affiliations:** ^1^College of Chinese Medicine, College of Integrated Chinese and Western Medicine, Nanjing University of Chinese Medicine, Nanjing, China; ^2^Independent Researcher, Boston, MA, United States; ^3^Chinese Medicine Centre, Western Sydney University, Campbelltown, NSW, Australia; ^4^NICM Health Research Institute, Western Sydney University, Westmead, NSW, Australia

**Keywords:** baicalin, antidepressant, corticosterone, neurogenesis, PI3K/AKT/GSK3β/β-catenin

## Abstract

Baicalin isolated from *Scutellaria baicalensis* possesses antidepressant abilities through its relation to hippocampal neurogenesis. Current research has found that baicalin can promote the proliferation of hippocampal granule cells, however, the detailed mechanism of baicalin on the survival and maturation of hippocampal granule cells has yet to be sufficiently explored. The purpose of this study was to evaluate whether baicalin could facilitate the survival and maturation of hippocampal granule cells, and to explore its potential mechanism. The chronic corticosterone (CORT)-induced mouse model of depression was used to assess antidepressant-like effects of baicalin and to illuminate possible molecular mechanisms by which baicalin affects hippocampal neurogenesis. The survival and maturation of granule cells were measured by immunohistochemistry, immunofluorescence and Golgi staining. The expression of Phosphatidylinositol 3-kinase (PI3K)/Protein kinase B (AKT)/glycogen synthase kinase-3β (GSK3β)/β-catenin pathway related proteins were measured by western blot analysis. PI3K inhibitor LY292002 and AKT inhibitor Perifosine were administered to HT-22 cells to explore the relationship between the PI3K/AKT/GSK3β/β-catenin pathway and baicalin. The results of the study illustrated that baicalin significantly decreased chronic CORT-induced depressive-like behaviors and reduced serum corticosterone levels. In addition, baicalin (administered at 60 mg/kg) reversed chronic CORT-induced lesions on hippocampal granule cells. Moreover, baicalin significantly increased the phosphorylation rate of PI3K, AKT, GSK3β, and total β-catenin. The study found that administration of LY292002/Perifosine counteracted the effects of baicalin in HT-22 cells. These results demonstrate that baicalin can alleviate chronic CORT-induced depressive-like behaviors through promoting survival and maturation of adult-born hippocampal granule cells and exhibiting protective effect on hippocampal neuron morphology. We propose the underlying mechanisms involve the activation of the PI3K/AKT/GSK3β/β-catenin pathway.

## Introduction

Depression is considered one of the most burdensome diseases globally, affecting approximately 264 million people worldwide (GBD 2017 Disease and Injury Incidence and Prevalence Collaborators, 2018). Major depressive disorder (MDD) is associated with an increased suicide risk and poor quality of life (Holden, 2005), accompanied by symptoms including: anhedonia, irritability, depressed mood, difficulty in concentrating, and abnormal appetite and sleep (Nakajima et al., 2010). Numerous studies have demonstrated that hippocampal atrophy and neurogenic damage in the adult hippocampus contribute to the pathogenesis of depression (Stratmann et al., 2014; Fenton et al., 2015; Kohman and Rhodes, 2017).

Recent studies show that corticosterone (CORT) levels are upregulated during stress, and hippocampal neurogenesis is inhibited by high CORT levels in primates (Bennett et al., 2002; Coe et al., 2003). Long-term exposure to CORT can induce depressive-like behaviors and cause neural deficits in the hippocampus of rodents (Alfarez et al., 2009; Morales-Medina et al., 2009), thus affecting neural progenitor cell differentiation, neuron survival, migration, and synaptic plasticity (Kunugi et al., 2010). Transgenic mice, with proapoptotic gene Bax deleted from neural stem cells, exhibit enhanced adult neurogenesis and resistance to depressive-like behaviors induced by CORT (Hill et al., 2015). Depressive-like behaviors induced by CORT can be improved by antidepressants including escitalopram, amitriptyline and fluoxetine (Caldarone et al., 2003; Furuse et al., 2019; Park, 2019). Antidepressants can accelerate the maturation of newborn hippocampal cells and promote their survival in animal models (Wang et al., 2008; Perera et al., 2011), resulting in decrease in depressive-like behavioral symptoms.

The PI3K/AKT/GSK3β/β-catenin signaling pathway involved in the survival and maturation of hippocampal granule cells plays an important role for promoting antidepressant effects (Chen et al., 2012; Matsuda et al., 2019). Activation of the PI3K pathway promotes PI3K-dependent phosphorylation and AKT phosphorylation. Downstream, the subsequent promotion of GSK3 phosphorylation inhibits GSK3 activity, thereby resulting in cytoplasm β-catenin enrichment. Downregulation of GSK3β activity promotes the stabilization and accumulation of β-catenin, increasing the availability of β-catenin to be transported to the nucleus, where it binds to transcription factors to upregulate transcription of neurodevelopmental genes (Carol and Richard, 2001; Isabel and Jeremy, 2001). Promotion of β-catenin enrichment facilitates the survival and maturation of DG neurons, thereby combatting depression and cognitive impairments, and contributing to antidepressant activities (Xing et al., 2016; Hui et al., 2018).

**Figure 1:**
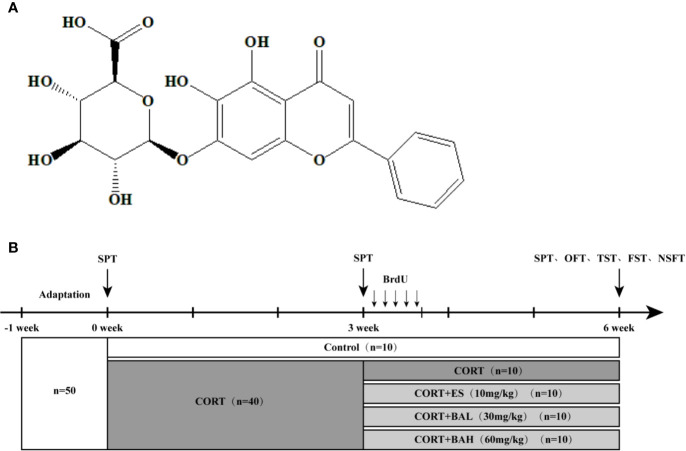
Chemical structure of baicalin and schematic representation of experimental groups and experimental procedures. **(A)** Chemical structural formula of baicalin; **(B)** The schematic representation of the experimental procedure.

Baicalin is a flavonoid isolated from the root of Scutellaria baicalensis (**Figure 1A**), and commonly used for treatment of depression in Chinese medicine. Previous studies have shown that baicalin can improve depressive-like behaviors induced by chronic unpredictable mild stress (CUMS), lipopolysaccharide (LPS) and CORT, and promote the proliferation of adult hippocampal granule cells (Zhang et al., 2016; Zhang et al., 2018; Guo et al., 2019). Moreover, research indicates that the PI3K/AKT signaling pathway is involved in baicalin’s antidepressant effect in LPS-induced depressive-like behavior in mice (Guo et al., 2019). Presently, the detailed mechanism of baicalin on the survival and maturation of hippocampal granule cells has yet to be sufficiently explored. The aim of this study was to evaluate whether baicalin could facilitate the survival and maturation of hippocampal granule cells and to detail its potential mechanism.

## Materials and Methods

### Animals and Experimental Procedure

Fifty adult female C57BL/6J mice (6~8 weeks, weighing 18–22 g) were purchased from the Shanghai Sipul-bikai Laboratory Animal Limited Company, and maintained under standardized environmental conditions consisting of: 12 h light/dark cycles (lights on from 6:00 a.m. to 6:00 p.m.), a constant temperature (22°C ± 2°C), food and water ad libitum, and five mice/cage. Animals were habituated to the standard conditions for one week, prior to experimental allocation and onset.

At baseline (week 0), mice were allocated into the following two groups: 1) untreated control (n=10) and 2) CORT paradigm (n=40). The procedure for establishing the mouse model of chronic CORT-induced depression was performed as previously described (Zhang et al., 2016). The 40 mice were subcutaneously injected daily for 6 weeks with CORT (40 mg/kg, TCI Development Co., Ltd, Japan) dissolved in 0.9% saline containing 0.1% dimethyl sulfoxide (DMSO). At the week-3 timepoint, CORT paradigm mice were allocated into the following four treatment groups (n=10 per group): CORT only, Escitalopram (ES, 10 mg/kg), Baicalin (BA 30 mg/kg), and Baicalin (BA 60 mg/kg). Baicalin (BA 30 and 60 mg/kg) and Escitalopram (ES, 10 mg/kg) were administered to mice in respective corresponding groups starting on the 22^nd^ day, on a daily frequency, until the end of the experiment (week 6). Baicalin (BA 30 and 60 mg/kg, Nanjing Liangwei Biotechnology Co., Ltd, China, purity >98%) was dissolved in 0.9% saline and given to mice daily by intragastric administration 30 min prior to the CORT injection. Escitalopram (ES, 10 mg/kg, Sigma, USA) was similarly dissolved in 0.9% saline and given to mice daily by intragastric administration 30 min prior to the CORT injection (**Figure 1B**).

To facilitate the study of cell dynamics, injections of BrdU (50 mg/kg/day, Sigma, USA) was administered intraperitoneally to mice in all groups for 5 days, starting on the 22^nd^ day, following the procedure advised in a previous study (Gao et al., 2018). Behavioral tests were performed at the end of the experiment (week-6), with sucrose preference training (SPT) also performed at baseline and week-3 timepoints, as it necessitates. The average body weight of animals was recorded for the six-week duration (see **Supplementary Figure 1**) and despite considerable body weight loss, no animals experienced mortality or were removed from the study due to CORT-related complications. All animals were euthanized on the second day following the behavioral tests’ completion. The experiment was approved by the Animal Care Committee of Nanjing University of Chinese Medicine. All efforts were made to minimize suffering and to reduce the number of animals used.

### Behavioral Tests

Five behavioral tests to assay anxiety- and depressive-like behaviors were conducted. Video tracking was used to record rodent behavior and to analyze the data. The behavioral tests were conducted following the procedure advised in a previous study (Zou et al., 2017) on days 42–44. The OFT and TST were performed 1 h after the last administration of CORT on day 42. The mice were then fasted (water and food) for 24 h. NFST was performed on day 43. After completion of NFST, SPT was performed, and mice were provided sufficient food and water. FST was performed on day 44 after completion of SPT.

#### Sucrose Preference Test

Mice received sucrose preference training for a period of 24 h upon which they received two bottles of sucrose solution (1%, w/v); followed by 48 h of sucrose adaptation where one of the bottles of sucrose solution was replaced with water, with bottle positions exchanged at every 24-h interval. After the process of sucrose adaptation, the mice were deprived of water for 24 h in individual cages. Following this, the mice were given free access to food and two bottles containing 100ml water and 100ml sucrose solution (1%, w/v). After 24 h, the volumes of consumed sucrose solution and water were recorded. Bottle positions were exchanged carefully every 12 h. Sucrose preference was calculated as percentage of the volume of sucrose solution consumed over the total volume of water consumed over the 24** h** of testing.

#### Open Field Test

Mice were individually placed in the center of an open field arena (40 cm × 40 cm ×40 cm) and allowed to explore freely for 5 min. The central area is defined as a square, sides 25 cm × 25 cm. A camera was mounted above the open box to record locomotor activity. The total distance traveled, and time spent in the central area were recorded and analyzed by the tracking system (DigBehavior, Shanghai, China).

#### Tail Suspension Test

Approximately 1** cm** from the end, each mouse**’**s tail was taped using adhesive tape, to a metal rod. Mice were suspended by the tail approximately 50 cm from the floor. A camera was mounted in front of the apparatus to record the movements of the mice for a total of 6 min. The immobility time was automatically detected and recorded with a tracking system during the last 4 min of the test.

#### Forced Swimming Test

Mice were individually placed in clean Plexiglas cylindrical tanks (height 25 cm, diameter 10 cm) filled to a depth of 10 cm with water (24°C ± 1°C) for 6 min. The movements of the mice were tracked with a mounted camera and the immobility time of the mice was recorded during the last 4 min of the test. Immobility was defined as absence of all movement except motions required to keep the mouse**’**s head above the water, as opposed to escape-seeking or exploratory behaviors.

#### Novelty-Suppressed Feeding Test

The mice were transferred to the experimental environment after fasting for 24 h. Each mouse was placed in the same corner of a new cage to explore for 10 min with a weighed food pellet placed in the center of the cage. The food consumption was recorded over 10** min**. Food consumption was calculated by the weight of food consumed/the weight of the mouse. The time the mice first started to consume the food was recorded as the latency to feed.

### Determination of Serum CORT Levels

Blood samples were collected after completion of the behavioral tests. The amount of time elapse between the last CORT injection and the measurement of serum CORT was approximately 55 h. Serum CORT levels were measured using commercial enzyme-linked immunosorbent assay (ELISA) kits in accordance with manufacturer’s instructions (ZCIBIO, China). The serum CORT of 10 mice per group was assayed, and the mean measurement was calculated as the final score for the group.

### Immunohistochemistry

Mice were anesthetized with sodium pentobarbital (50 mg/kg, intraperitoneally) and were transcardially perfused (saline, followed by 4% paraformaldehyde in PBS). Brains were obtained and post-fixed overnight in 4% paraformaldehyde at 4°C, and then transferred to 20% and 40% sucrose solution. Serial sections of 30 μm thickness were cut through the entire hippocampus using a cryostat and stored in cryoprotectant (50% glycerin in PBS). Every sixth coronal section from the adult dentate gyrus was used to assess Ki67 and BrdU positive cells in both hemispheres (10 sections per animal, 3 mice per group). The sections were washed by PBS, then put into 3% hydrogen peroxide for 15 min at room temperature. After three washes by PBS, the sections were sealed by 10% BSA (0.3% triton-x100/PBS) at room temperature for 1 h. The sealing fluid was absorbed, and the sections were incubated for 24 h at 4°C with primary antibody rabbit anti-Ki67(1:200, ab15580, Abcam) or anti-BrdU (1:200, ab6326, Abcam). The tissues were washed in PBS and then subsequently incubated with goat anti-rabbit hrp-labeled secondary antibody (1:2000, ab205718, Abcam) for 40–60 min at room temperature. DAB staining was applied to the brain sections, color observation was made against white paper, and the stain process was concluded within an appropriate time. Ki67 and BrdU positive cells were observed and counted under an Olympus BX63 microscope (Olympus, Tokyo, Japan) fitted with a ×10 eyepiece lens with a ×40 objective.

### Immunofluorescence

A triple-staining procedure was implemented for BrdU/DCX/NeuN to allow analyzation through immunofluorescence. The brain sections were incubated overnight at 4°C with the following primary antibodies: rat anti-BrdU (1:200, ab6326, Abcam), mouse anti-NeuN (1:500, ab104224, Abcam) and rabbit anti-DCX (1:500, ab18723, Abcam). The sections were washed in PBS and incubated for 4 h with fluorescent secondary antibodies: Alexa Fluor 488 goat anti-rat IgG to reveal immunoreactivity of BrdU, Alexa Fluor 405 donkey anti-mouse IgG to reveal immunoreactivity of NeuN, and Alexa Fluor 594 goat anti-rabbit IgG to reveal immunoreactivity of DCX, respectively (1:400 for all three antibodies, Abcam). Every sixth coronal section from the adult dentate gyrus was used to assess BrdU+/NeuN+/DCX- and BrdU+/NeuN+/DCX+ positive cells in both hemispheres (10 sections per animal, 3 mice per group). BrdU+/NeuN+/DCX- and BrdU+/NeuN+/DCX+ positive cells were observed and counted under an Olympus BX63 microscope fitted with a ×10 eyepiece lens with a ×40 objective, and their percent distribution in the BrdU positive cells was calculated.

### Golgi Staining

Golgi staining was performed according to the manufacturer’s instructions (Hito Golgi-Cox OptimStainTM Kit, HTKNS1125, Hitobiotec Corp., USA). Freshly dissected brains were immersed in a mixture containing potassium dichromate and chromate at 25°C for 2 weeks and then transferred into Solution 3 according to instruction kit protocol, where they rested at 4°C for 72 h in a light-deprived area. Brain samples were then cut into 150-μm sections and transferred to the gelatin-coated slides. Samples were clarified with xylene and fixed with Neutral Balsam (Beijing Solibao Technology Co. Ltd, China). Morphological analysis was performed on pyramidal cells in accordance with the established criteria (Li et al., 2004) using ImageJ 6.0 and an Olympus BX63 microscope (Olympus, Tokyo, Japan).

### Cell Culture

The HT-22 cells were cultured in Dulbecco’s Modified Eagle Medium (DMEM, Gibco) supplemented with 10% fetal bovine serum (FBS, Biological Industries) and incubated at 37 °C in a humidified incubator under 5% carbon dioxide. The cells were pretreated with baicalin (30 μM), LY292002 (20 μM, HY-10108, MCE) or Perifosine (40 μM, HY-60909, MCE) for 1 h followed by post-incubation with CORT (600 μM) for 24 h, and cell viability was measured by MTT assay.

### Western Blot

The samples were lysed in a RIPA buffer containing protease inhibitors and phosphatase inhibitors. The protein concentration was determined by the BCA method (Pierce, Rockford, IL, USA), and the protein cleavage product was separated by 10% SDS-PAGE electrophoresis and transferred to a polyvinylidene fluoride (PVDF) membrane. After blocking with 5% BSA for 1 h, the membrane was incubated with a primary antibody at 4°C overnight. Primary antibodies include PI3K (1:1,000, 11889, Cell Signaling), Phospho-PI3K (1:1000,4228, Cell Signaling), AKT (1:500, 10176-2-A, Proteintech), Phospho-AKT (1:500,66444-1-lg, Proteintech), GSK-3β (1:1,000, 9315, Cell Signaling), Phospho-GSK-3β (Ser9) (1:1,000, 5558, Cell Signaling), β-catenin (1:1,000, ab32572, Abcam), PSD95(1:1,000, 2507, Cell Signaling), Synaptophysin 1(1:1000,4329, Cell Signaling). After incubation with the appropriate secondary antibody for 1 h, the blot was visualized using SuperSignal West Pico Chemiluminescent Substrate (Thermo Fisher Scientific Inc.). The optical density was quantified by Image-J 6.0.

### Statistical Analysis

According to the D’Agostino & Pearson or Shapiro–Wilk normality test, our data exhibited a Gaussian distribution. T-tests and one-way analysis of variance (ANOVA) followed by Dunnett’s test were carried out using Graphpad 6.0, correcting for multiple comparisons. T-tests were carried out to analyze the results of SPT and body weight at the first three weeks, while one-way ANOVA was used to determine the results of all the other assessments. Statistical analysis results were expressed as mean ± SEM, with a P<0.05 considered statistically significant.

## Results

### Baicalin Decreases Chronic CORT-Induced Depressive-Like Behaviors and Serum CORT

**Figure 2:**
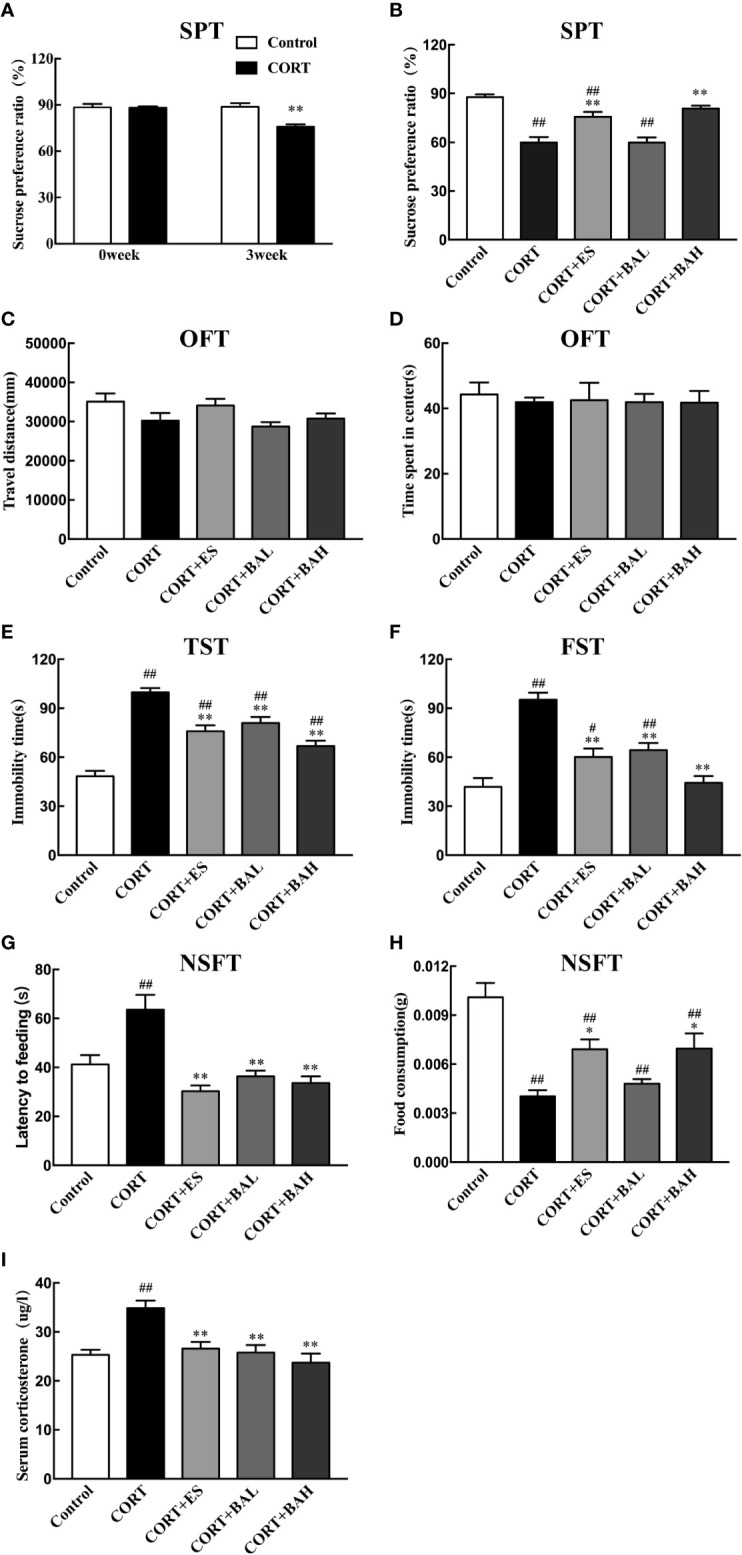
Effect of baicalin on different behaviors and serum corticosterone in corticosterone (CORT) induced depression model mice. **(A)** The basal sucrose preference and the changes after three weeks of CORT paradigm; **(B)** The effect of baicalin on sucrose preference in CORT-induced depression model mice; **(C, D)** Performance in the open field test; **(E)** Immobility time in the tail suspension test; **(F)** Immobility time in the forced swimming test; **(G, H)** Performance in Novelty-suppressed feeding test; **(I)** The effect of baicalin in the changes of serum corticosterone. Data is expressed as mean  ± SEM (n=10 mice/group). ^#^P < 0 .05 and ^##^P  <  0.01 versus Control, ^*^P < 0.05 and ^**^P < 0.01 versus CORT.

The chronic CORT-induced depression model in mice was used to study the antidepressant effects of baicalin (30, 60 mg/kg), and its comparison to escitalopram (10 mg/kg). In the SPT experiment, there was no difference at baseline in sucrose consumption between the untreated control and CORT paradigm group (*t*=0.02, P>0.05) (**Figure 2A**). At the week-3 time point, sucrose preference was significantly decreased in the CORT paradigm group (*t*=3.98, P<0.01) (**Figure 2A**), indicating that the CORT paradigm was successfully established. At the week-6 timepoint, there was significant increase in sucrose preference in both baicalin (60 mg/kg) and escitalopram (10 mg/kg) groups compared to the CORT only [F(4,45)=22.44, P<0.01, **Figure 2B**], and sucrose preference in the baicalin (60 mg/kg) group was close to control (P>0.05). There was no statistical difference between groups in total distance traveled [F(4,45)=2.57, P>0.05, **Figure 2C**] and central time [F(4,45)=0.26, P>0.05, **Figure 2D**] in the OFT experiment, indicating that chronic CORT exposure did not affect autonomous activities. In the TST and FST experiments, immobility time in the CORT only group was significantly increased [F(4,45)=32.44, P<0.01, **Figure 2E**; F(4,45)=20.4, P<0.01, **Figure 2F**], while immobility time in the baicalin (30, 60 mg/kg) and escitalopram (10 mg/kg) groups were significantly decreased compared to the CORT only group (P<0.01), and immobility time of the baicalin (60 mg/kg) in FST were close in range to the control group (P>0.05). In the NSFT experiment, feeding latency was increased [F(4,45)=20.22, P<0.01, **Figure 2G**] and food consumption was decreased [F(4,45)=12.63, P<0.01, **Figure 2H**] in the CORT only group, while the opposite was found in both baicalin (60 mg/kg) and escitalopram (10 mg/kg) groups compared to CORT only group (P<0.01; P<0.05, respectively). There was significant reduction in food consumption across CORT+ES, CORT+BAL and CORT+BAH groups compared to the control (P<0.01 respectively). The plasma CORT levels in the CORT only group were significantly increased [F(4,45)=10.73, P<0.01, **Figure 2I**], and were significantly decreased in the baicalin (30, 60 mg/kg) and escitalopram (10 mg/kg) groups (P<0.01) compared to CORT only group. The results of the behavioral studies indicate the effectiveness of baicalin in improving chronic CORT-induced depressive-like behaviors and reducing plasma CORT levels in mice, with comparable results to the established antidepressant escitalopram.

### Baicalin Increases Proliferation and Survival of Newborn Cells in DG

**Figure 3:**
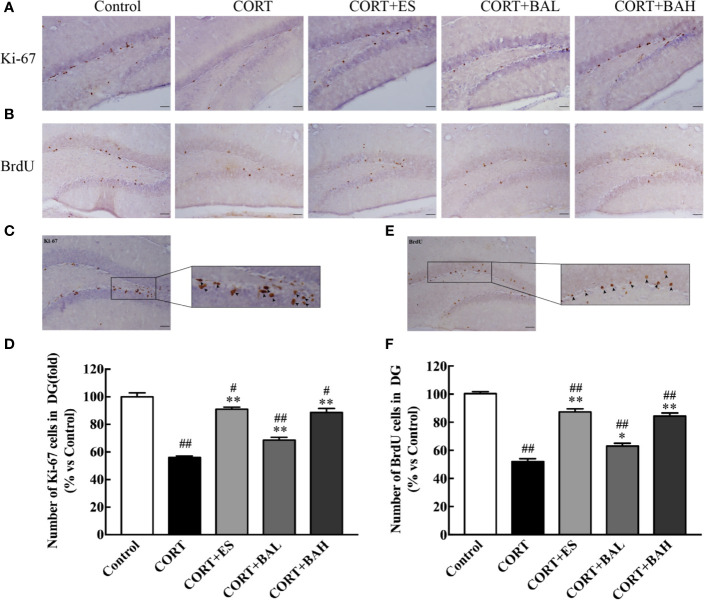
Baicalin treatment increased proliferation and survival of newborn granule cells in the dentate gyrus of chronic corticosterone (CORT)-induced depression model mice. **(A)** Images of Ki-67 immunohistochemistry in DG granular neurons; **(B)** Images of BrdU immunohistochemistry in DG granular neurons; **(C)** Enlarged image of the Ki-67 cells; **(D)** The total number changes of Ki-67 cells; **(E)** Enlarged image of the BrdU cells; **(F)** The total number changes of BrdU cells. The results are mean  ± SEM (n=3 mice/group), scale bar=50 μm. ^#^P < 0 .05 and ^##^P  <  0.01 versus Control, ^*^P < 0.05 and ^**^P < 0.01 versus CORT.

Immunohistochemistry was used to detect the effect of baicalin on neurogenesis after chronic CORT exposure, with brain sections assessed by Ki67 tracking. The results showed a significant reduction in neurogenesis after chronic exposure to CORT [F(4,10)=66.92, P<0.01, **Figures 3A, D**], whereas treatment with baicalin (30, 60 mg/kg) and escitalopram (10 mg/kg) significantly increased the number of Ki-67 positive cells (P<0.01) compared to CORT only. The enlarged Ki-67 positive cells are shown in **Figure 3C**. This suggests that baicalin can promote neurogenesis in chronic CORT-induced depression model mice. Furthermore, as shown in **Figures 3B, F**, the results of BrdU immunohistochemistry indicated that newborn granule cells survival in DG was significantly reduced after chronic exposure to CORT [F(4,10)=93.76, P<0.01], while treatment with baicalin (30, 60 mg/kg) and escitalopram (10 mg/kg) significantly increased cell survival (P<0.05, P<0.01 and P<0.01respectively) compared to CORT only. The enlarged BrdU positive cells are shown in **Figure 3E**. There was significant reduction in Ki-67 positive cells in CORT+ES, CORT+BAL and CORT+BAH groups compared to the control (P<0.05, P<0.01, and P<0.05 respectively), and significant reduction in BrdU positive cells across the three treatment groups compared to the control (P<0.01 respectively). The results suggest that baicalin prevented CORT-induced changes in proliferation and survival of newborn granule cells but did not normalize the changes.

### Baicalin Promotes Maturation of Newborn Granule Cells in DG

**Figure 4:**
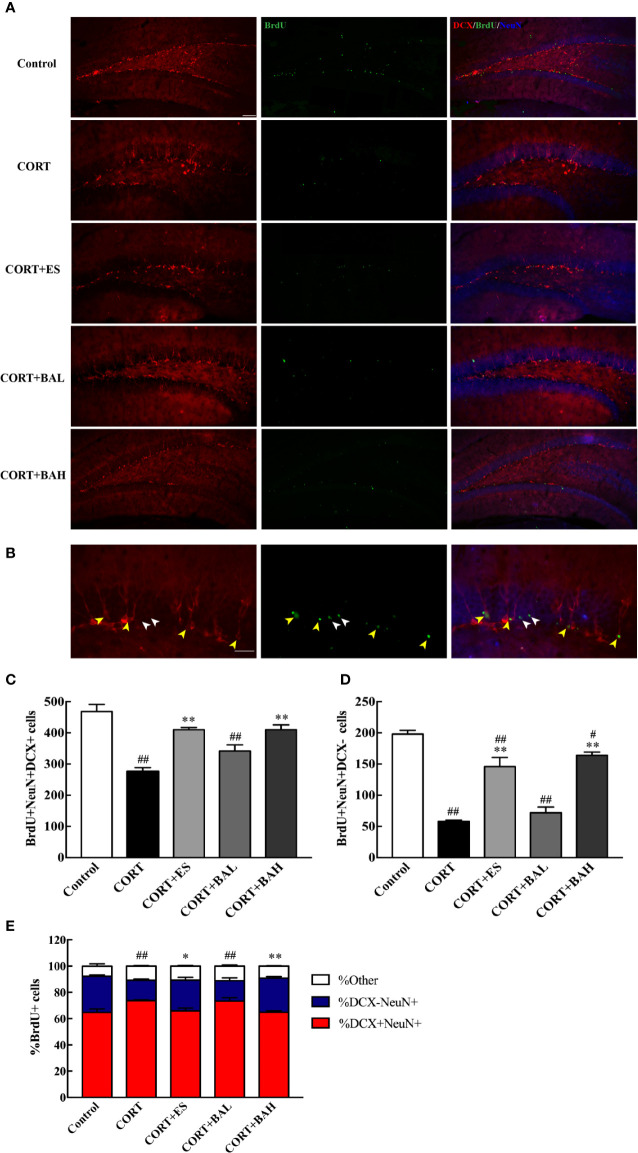
Baicalin promotes maturation of newborn granule cells in the dentate gyrus of chronic corticosterone (CORT)-induced depression model mice. **(A)** Hippocampal dentate gyrus (DG) images of the adult newborn neurons (red: DCX; green: BrdU; Blue: NeuN), scale bar=50 μm; **(B)** Enlarged images of the newborn immature neurons (morphology of DCX+/NeuN+/BrdU+ cells, yellow arrows) and the mature neurons (morphology of DCX-/NeuN+/BrdU+ cells, white arrows), scale bar=20 μm; **(C)** The number of the newborn immature neurons; **(D)** The number of adult born mature neurons; **(E)** The proportion of DCX-/NeuN+/BrdU+ cells (percentage of BrdU cells). The results are mean  ± SEM (n=3 mice/group). ^#^P < 0 .05 and ^##^P < 0.01 versus Control, ^*^P<0.05 and ^**^P<0.01 versus CORT.

To explore the effect of baicalin on the maturation of newborn granule cells, triple-staining immunofluorescence of BrdU, DCX and NeuN markers was analyzed (**Figure 4A**). The number of BrdU+/DCX+/NeuN+(**Figure 4B**, yellow arrows) and BrdU+/DCX-/NeuN+(**Figure 4B**, white arrows) positive cells decreased after CORT exposure [F(4,10)=20.33, P<0.01, **Figure 4C**; F(4,10)=47.53, P<0.01, **Figure 4D**], while cell numbers increased after treatment with baicalin (60 mg/kg) and escitalopram (10 mg/kg) (P<0.01) compared with CORT only. BrdU+/DCX-/NeuN+ positive cells in baicalin (60 mg/kg) and escitalopram (10 mg/kg) were decreased compared to control group respectively (P<0.05; P<0.01, respectively). In order to observe the maturation of the BrdU cells in each group, the respective ratios of the different cells tested were found and analyzed. The results showed that the ratio of mature neurons decreased after chronic CORT exposure [F(4,10)=14.11, P<0.01, **Figure 4E**], while their ratios increased after the treatment of baicalin (60 mg/kg) and escitalopram (10 mg/kg) (P<0.05; P<0.01, respectively) compared to CORT only. When combined, these results show the positive influence of baicalin in promoting the maturation of newborn granule cells.

### Baicalin Enhances Dendritic Complexity of Neuron Cells in DG

**Figure 5:**
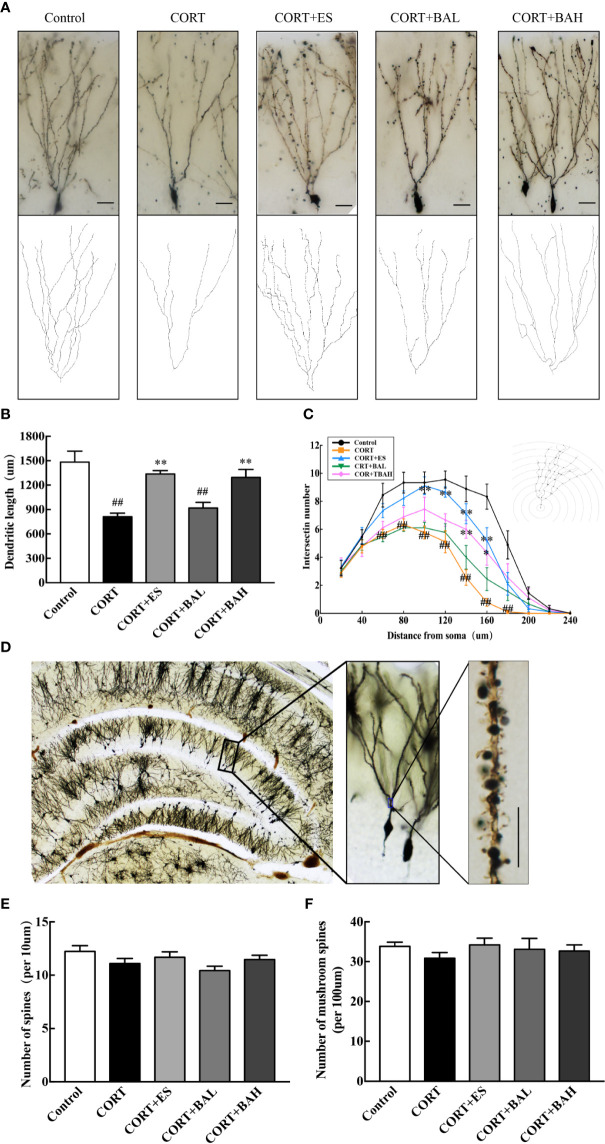
Baicalin enhances dendritic complexity without affecting spine density of granule cells in the dentate gyrus of chronic corticosterone (CORT)-induced depression model mice. **(A)** Dendritic arbors measured by Golgi staining and reconstruction of the dendritic arbors in DG granular neurons, scale bar=50 μm; **(B)** Sholl analyses of total dendritic length in DG granule neurons; **(C)** Sholl analyses of the numbers of dendritic points in DG granule neurons; **(D)** Representative graphs of dendritic spines in hippocampal DG granule neurons, scale bar =10 μm; **(E)** The density of the total spines; **(F)** The density of the mushroom-shaped spines. Data is represented as means ± SEM (n=3 mice/group, **(B, C)** 3 neurons were analyzed for each animal; **(E, F)** 10 neurons were analyzed for each animal). ^##^P  <  0.01 versus Control, ^*^P < 0.05 and ^**^P < 0.01 versus CORT.

Golgi staining was used to detect the synaptic dendrite complexity, spine density and the morphology of DG neurons (**Figures 5A, D**). Following the experiment, the total dendritic length of DG granule neurons were significantly reduced after chronic exposure to CORT [F(4,40)=15.83, P<0.01, **Figure 5B**], while escitalopram (10 mg/kg) and baicalin (60 mg/kg) treatment normalized this structural change (P<0.01). Meanwhile, CORT exposure also can significantly reduce the total intersections of the DG granule neurons [F_60_(4,40)=5.67, F_80_(4,40)=6.04, F_100_(4,40)=6.52, F_120_(4,40)=10.54, F_140_(4,40)=11.15, F_160_(4,40)=13.04, F_180_(4,40)=4.94, P<0.01, **Figure 5C**], while escitalopram (10 mg/kg) and baicalin (60 mg/kg) can increased the total intersections compared to the CORT only, especially at the distance of 140–160 μm (P<0.01 for 140 μm; P<0.01, P<0.05 respectively for 160 μm). Spine density and mushroom spine density, measured at a distance of 100–160 μm from the soma in DG neurons, was unaltered by chronic exposure to CORT, and similarly unaltered following baicalin treatment [F(4,145)=2.02, *P*>0.05, **Figure 5E**; F(4,145)=0.55, *P*>0.05, **Figure 5F**]. The results indicate that chronic CORT exposure induced dendritic atrophy without impacting spine density and mushroom spine density, while baicalin treatment normalized the structural change caused by chronic CORT-exposure, and similarly did not impact spine density and mushroom spine density in granule cells.

### Baicalin Attenuates the Depressive-Like Behaviors Through Activation of PI3K/AKT/GSK 3β/β-Catenin Pathway

**Figure 6:**
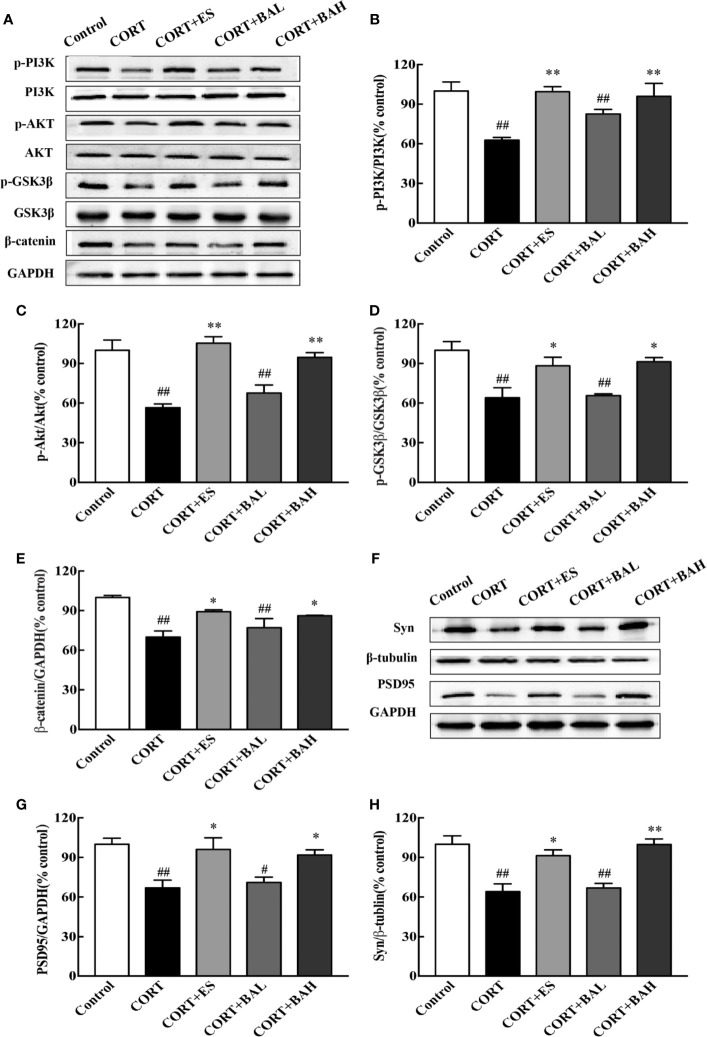
PI3K/AKT/GSK3β/β-catenin pathway is involved in the antidepressant effect of baicalin. **(A)** Representative western blots of PI3K/AKT/GSK3β/β-catenin pathway relative protein expression; **(B–E)** The quantification of p-PI3K, p-AKT, p-GSK-3β (Ser9) and β-catenin protein expression in hippocampus; **(F)** Representative western blots of PSD95 and Synaptophysin1 protein expression in hippocampus; **(G, H)** The quantification of PSD95 and Synaptophysin1 protein expression in hippocampus. Data are represented as means ± SEM (n=3 mice/group). ^#^P  < 0 .05 and ^##^P  <  0.01 versus Control, ^*^P < 0.05 and ^**^P < 0.01 versus CORT.

To investigate whether the PI3K/AKT/GSK3β/β-catenin pathway was involved in the antidepressant effect of baicalin, western blot analysis was performed to assess the expression of related proteins in the hippocampus. We found that the p-PI3K, p-AKT, p-GSK3β, and β-catenin protein expression levels were significantly decreased after CORT exposure [F(4,10)=7.23, F(4,10)=16.28, F(4,10)=8.49, F(4,10)=8.81, P<0.01, **Figures 6A–E**], and these changes were normalized after escitalopram (10 mg/kg) and baicalin (60 mg/kg) treatment (P<0.01 for p-PI3K and p-AKT; P<0.01, P<0.05 for p-GSK3β and β-catenin) compared to CORT only. Next, protein expression levels of synaptic proteins PSD95 and Syn in the hippocampus were analyzed. As shown in **Figures 6F–H**, the total PSD95 and Syn expression were significantly decreased in mice after chronic CORT exposure [F(4,10)=7.12, F(4,10)=12.55, P<0.01, **Figures 6G, H**], while these levels were significantly increased after treatment with escitalopram (10 mg/kg) and baicalin (60 mg/kg) (P<0.05 for PSD95; P<0.05, P<0.01 respectively for Syn) compared to CORT only.

**Figure 7:**
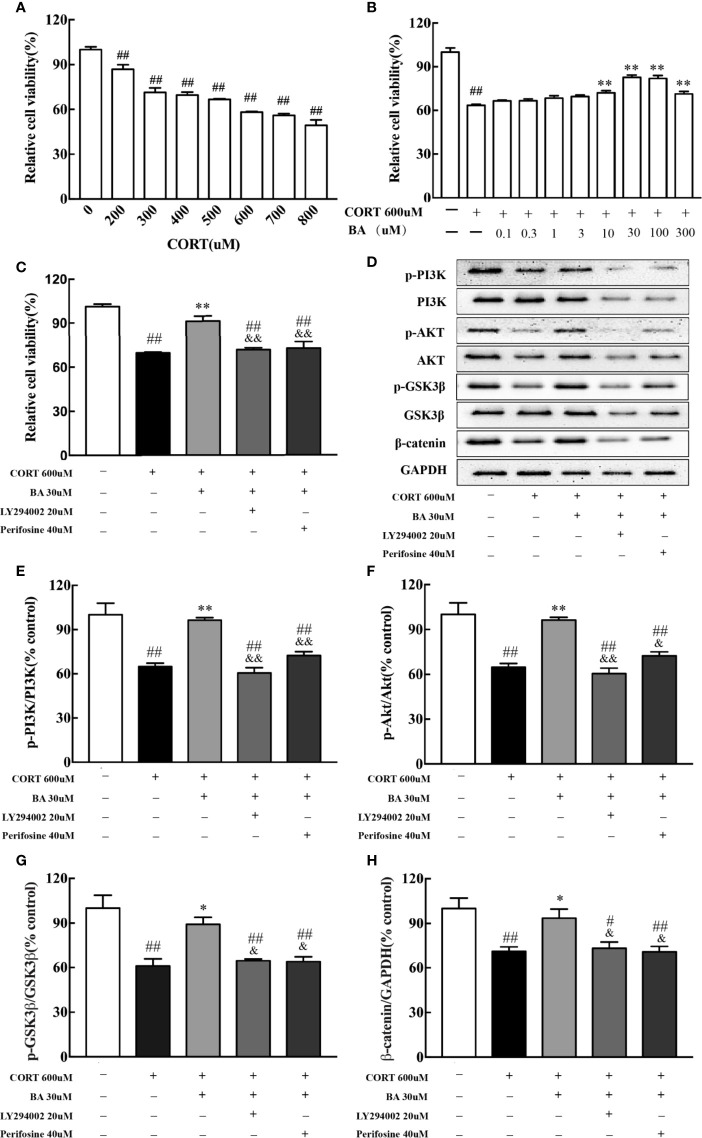
LY294002 and Perifosine blocked the effect of baicalin in HT-22 cells induced by corticosterone (CORT). **(A)** The effect of corticosterone induced cell viability loss in HT-22 cells; **(B)** Baicalin alleviates corticosterone-induced cell viability loss in HT-22 cells; **(C)** PI3K inhibitor LY294002 and AKT inhibitor Perifosine blocked the effect of baicalin; **(D)** Representative western blots of PI3K/AKT/GSK3β/β-catenin pathway relative protein expression; **(E–H)** The quantification of p-PI3K, p-AKT, p-GSK-3β (Ser9), and β-catenin protein expression in CORT-induced HT-22 cells. Data are represented as means ± SEM (Each result based on three times experiment). ^#^P  < 0 .05 and ^##^P  <  0.01 versus Control, ^*^P < 0.05 and ^**^P < 0.01 versus CORT, ^&^P < 0.05 and ^&&^P < 0.01 versus CORT+BA.

*In vitro*, HT-22 cells were pretreated by baicalin for 1 h. Cell viability was determined by MTT assay. The drug concentration for the best CORT model of HT-22 cells was 600uM [F(7,16)=52.96, P<0.001, **Figure 7A**], while the optimal concentration of baicalin, LY294002 and Perifosine was 30 μM, 20 μM, and 40 μM respectively [F(9,20)=32.06, F(4,10)=26.26, P<0.01, **Figures 7B, C**]. After 24 h of CORT exposure, the protein levels of p-PI3K, p-AKT, p-GSK3β, and β-catenin were significantly decreased [F(4,10)=18.48, F(4,10)=18.40, F(4,10)=11.59, F(4,10)=7.90, P<0.01, **Figures 7D–H**] compared to control, and were significantly up-regulated by baicalin treatment (P<0.01 for p-PI3K and p-AKT; P<0.05 for p-GSK3β and β-catenin) compared to CORT only. Interestingly, the effect of baicalin was counteracted in the LY294002 and Perifosine treated groups (P>0.05) compared to CORT only. These results suggest that baicalin may improve depressive-like behaviors through its activation of PI3K/AKT/GSK3β/β-catenin pathway.

## Discussion

Numerous studies have illustrated baicalin’s neuroprotective abilities in central nervous system diseases (Zhang et al., 2016; Gao et al., 2018; Sowndhararajan et al., 2018; Zhang et al., 2018; Guo et al., 2019; Jin et al., 2019). In addition, prior studies have demonstrated that baicalin has antidepressant effects and can reduce neuroinflammation (Guo et al., 2019; Jin et al., 2019), protect neuron apoptosis (Zhang et al., 2018) and promote adult hippocampal neurogenesis in animal models of depression (Gao et al., 2018). Although current research indicates that baicalin can promote the proliferation of hippocampal granule cells, the mechanism of baicalin on the survival and maturation of hippocampal granule cells has yet to be sufficiently detailed.

Corresponding to prior literature, the chronic CORT administration used in the present study established a preclinical model of depression, through the induction of behavioral and neurological changes that are consistent in patients with depression (Zhao et al., 2009; Sterner and Kalynchuk, 2010; Rainer et al., 2012). In this study, following 6 weeks of subcutaneous injection of CORT, mice exhibited significant behavioral alterations including: decreased sucrose preference, increased immobility time in FST and TST, increased latency and decreased food intake in NFST, and increased serum CORT level. These results are indicative that subcutaneous injections of CORT induce depressive-like behaviors in female mice. Complementing previous studies of baicalin’s ability to decrease chronic CORT-induced depressive-like behaviors in male mice (Li et al., 2015; Gao et al., 2018), this study found that baicalin has the same capabilities for female mice under the same preclinical model of depression, thus enhancing the evidence of baicalin’s potential for antidepressant effects.

Recently, the correlation between granule cell proliferation and their antidepressant effects have been documented (Park, 2019). Increased neurogenesis achieved by improving the survival rate and maturation of newborn neurons can decrease depressive-like behaviors (Sterner and Kalynchuk, 2010; Anacker et al., 2018). While previous studies have found that baicalin could increase the proliferation of adult granule cells in animal models of depression (Zhang et al., 2016; Gao et al., 2018), its effect on the survival and maturation of proliferated granule cells required further investigation. In order to do this for the present study, BrdU labeling was conducted at the start of baicalin administration. Following its completion, it was found that after CORT exposure, the number of Ki67 labeled proliferating cells was reduced, which is consistent with previous research (Anacker et al., 2018), while the effect was countered by baicalin treatment, as the number of Ki67 positive cells significantly increased, and were normalized closer to the control. The number of BrdU cells in the DG was also significantly increased after baicalin treatment, and it was shown that baicalin facilitated the maturation of newborn granule cells and increased the number of BrdU+/NeuN+/DCX− neurons. The evidence of these results reinforces baicalin’s abilities in promoting the survival and maturation of granule cells.

xChanges in hippocampal neuron morphology after baicalin treatment were assessed in this study. Previous studies showed that chronic CORT exposure caused decreased neurogenesis and impaired dendrite morphology in DG and CA regions, and depression-related behavioral symptoms (Watanabe et al., 1992; Kim and Diamond, 2002; Bechstedt et al., 2014). Studies have also found that dendritic structure and complexity in the basal dendrites of CA3 pyramidal neurons was reduced by postpartum CORT administration (Sousa et al., 2000; Workman et al., 2013). Consistent to prior findings, this study illustrated that the six-week CORT paradigm induced dendritic atrophy without affecting spinal density in granule cells and found that baicalin significantly improved the dendritic complexity of granule cells and without altering the morphology of the mushroom spines of basal dendrites in DG. Moreover, CORT-induced downregulation in protein expressions of the presynaptic marker (synaptophysin1) and the postsynaptic marker (PSD95) was blocked following baicalin treatment. These results suggest that baicalin may also play a protective role in hippocampal neuron morphology.

Recent studies of mice models of depression have indicated that changes in synaptic plasticity, learning, memory, and depression are related to issues in the PI3K signaling pathway (Matsuda et al., 2019). Activation of PI3K pathway promotes both AKT and subsequent GSK3β phosphorylation, which then inhibits the latter’s activity. Downregulation of GSK3β through its inhibition and/or decreased activity promotes the stabilization and accumulation of available cytoplasmic, and ultimately nucleic β-catenin, where it can regulate and promote neurodevelopmental genes. Therefore, decreased GSK3β activity and subsequent β-catenin enrichment aids in the promotion of efficient formation of microtubules throughout the neurite, resulting in more branching and synaptic connections in adult-born granular neurons (Garrido et al., 2007; Llorens-Martin et al., 2016). β-catenin, the main substrate of GSK3β, has been identified as a key regulator of depressive-like behaviors (Madsen et al., 2003; Dias et al., 2014), and increased GSK3β activity was correlated with decreased levels of β-catenin in the prefrontal cortex of depressed patients (Karege et al., 2012). Our previous experiments showed that baicalin increased the expression of phosphorylated GSK3β (Ser9) protein and activation of PI3K/AKT pathway in the hippocampus of CUMS model animals (Zhang et al., 2018; Guo et al., 2019). The current data showed that chronic CORT exposure reduces the expression of phosphorylated PI3K, AKT, GSK3β (Ser9) proteins and total β-catenin protein, while baicalin treatment could counter CORT effects. To confirm the crucial role of baicalin-regulated PI3K/AKT/GSK3β/β-catenin pathway in regulating hippocampal granule cells, inhibitor validation treatment was performed on the hippocampal neurons of HT-22 cells. We found that the effect of baicalin on CORT-induced HT-22 cells was counteracted by PI3K and AKT inhibitor. These results suggest that baicalin and CORT produce opposing effects on adult-born hippocampal granule cells, particularly through baicalin’s activation of the PI3K/AKT/GSK3β/β-catenin pathway. Hence, baicalin may combat depression and depression-related cognitive impairments by facilitating survival and maturation of DG neurons through upregulation of β-catenin enrichment.

**Figure 8:**
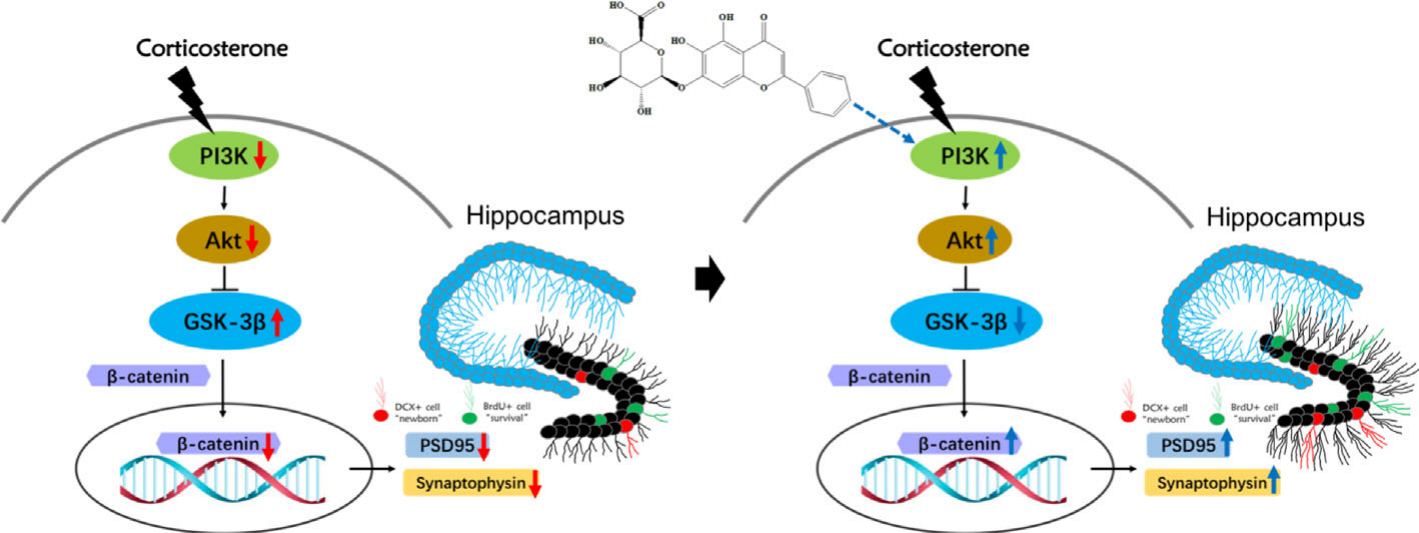
Schematic illustration of the mechanism for baicalin’s promotion of hippocampal granule cells survival and maturation in corticosterone (CORT)-induced depression model mice. In the normal physiological state, activation of PI3K promotes both AKT and subsequent GSK3β phosphorylation of Ser 9, which then inhibits the latter’s activity. The decreased activity of GSK3β promotes the stabilization and accumulation of nucleic β-catenin, where it can regulate and promote neurodevelopmental genes related to survival and maturation of granule cells. Prolonged exposure to high corticosterone reduces the expression of phosphorylated PI3K, AKT, GSK3β (Ser9) proteins and total β-catenin protein, resulting in decreased branching and synaptic connections in adult-born granular neurons. Baicalin can activate the PI3K/AKT/GSK3β/β-catenin pathway, facilitating survival and maturation of DG granular cells.

In conclusion, this study demonstrated that baicalin effectively alleviated chronic CORT induced depressive-like behaviors in mice and promoted hippocampal neurogenesis. The effect of baicalin may be attributed to its ability to promote survival and maturation of adult-born hippocampal granule cells, and its protective effect on hippocampal neuron morphology. The underlying mechanisms involve baicalin’s related activation of the PI3K/AKT/GSK3β/β-catenin pathway (**Figure 8**): its counteractive abilities on CORT, and its promotion of the PI3K/AKT enzymatic signaling pathway proteins, including enrichment of neurodevelopment transcription regulator β-catenin. These results support the interest of baicalin-based therapeutic strategies, and to further the development of complementary medicines for treating depression.

## Data Availability Statement

The raw data supporting the conclusions of this article will be made available by the authors, without undue reservation.

## Ethics Statement

The animal study was reviewed and approved by the Animal Care Committee of Nanjing University of Chinese Medicine.

## Author Contributions

FZ, WT, and RQ designed the experiments. FZ, WT, ZS, and WZ performed research and analyzed data. JR, CZ, HA, HL, and LZ provided materials. FZ, WT, HA, HL, and RQ wrote the manuscript. All authors contributed to the article and approved the submitted version.

## Funding

This research was funded by National Natural Science Foundation of China (No. 81573701, 81873096), Postgraduate Research & Practice Innovation Program of Jiangsu Province (KYCX19_1282) and the Priority Academic Program Development of Jiangsu Higher Education Institutions (Integration of Chinese and Western Medicine).

## Conflict of Interest

The authors declare that the research was conducted in the absence of any commercial or financial relationships that could be construed as a potential conflict of interest.
