# Supplementary material for: Facilitating Granule Cell Survival and Maturation in Dentate Gyrus With Baicalin for Antidepressant Therapeutics
Source: Front Pharmacol. 2020 Sep 2;11:556845. doi: 10.3389/fphar.2020.556845 (PMC7493074; doi:10.3389/fphar.2020.556845)
Supplement: Supplementary file 1 [file DataSheet_1.docx]

Supplementary Material

# Supplementary Figures


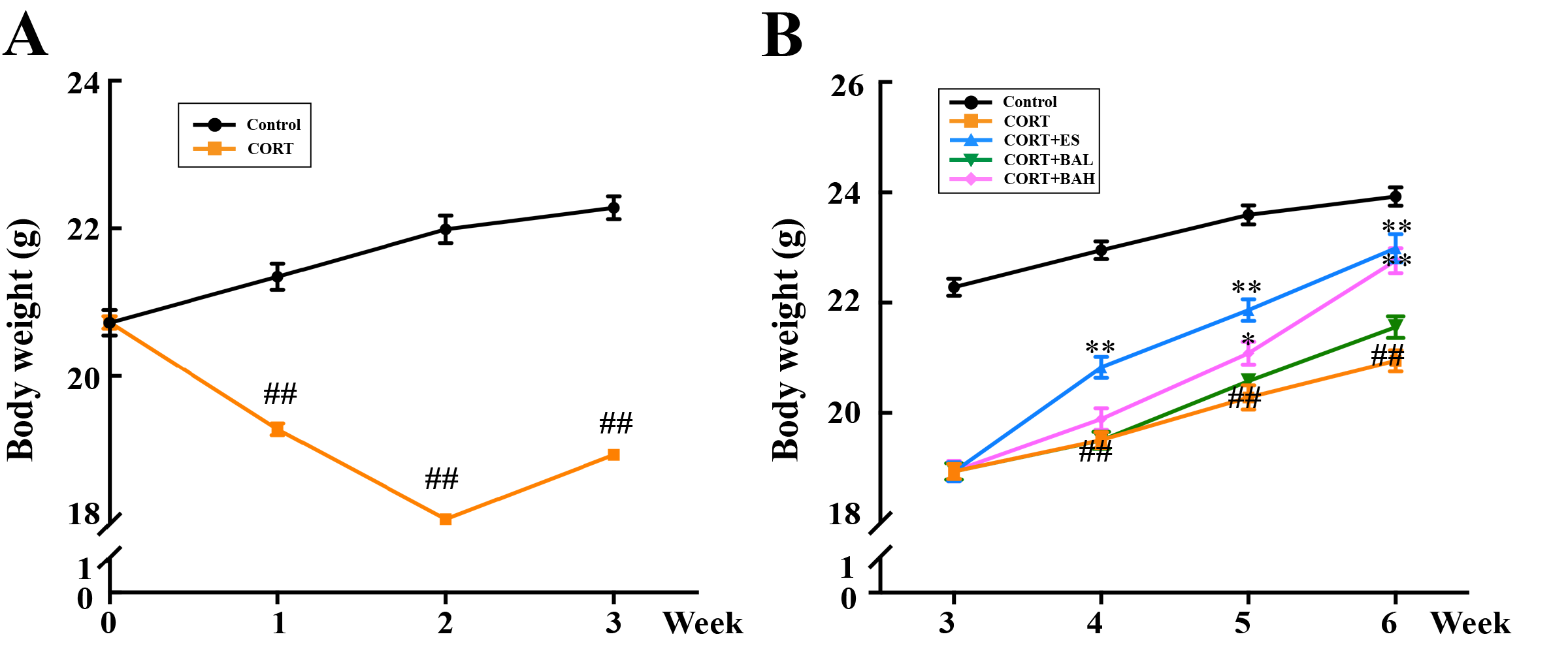


**Supplementary Figure 1. Effect of corticosterone** **and baicalin on body weight.** A. The basal body weight and the changes after three weeks of CORT paradigm. B. The effect of baicalin on body weight in CORT-induced depression model mice. Data are represented as means±SEM (n=10 mice/group). ^#^P <0 .05 and ^##^P < 0.01 versus Control, ^*^P<0.05 and ^**^P<0.01 versus CORT.
